# GERIATRIC URINARY INCONTINENCE AND LOWER URINARY TRACT SYMPTOMS: NEW DATA, TRIALS, AND CLINICAL APPROACHES

**DOI:** 10.1093/geroni/igae098.2047

**Published:** 2024-12-31

**Authors:** Scott Bauer, George Kuchel

**Affiliations:** Chair; Discussant

## Abstract

Lower urinary tract symptoms (LUTS) is a syndrome of commonly overlapping symptoms that occur when urine is being generated and stored in the bladder, called storage LUTS (e.g., overactive bladder and urinary incontinence), or during the initiation and process of urination, called voiding LUTS (e.g., weak stream). More than 1 out of 3 of older adults report clinically significant LUTS, contributing to poor quality of life and increased risk of falls and fractures, functional and mobility impairment, and death. Although urinary incontinence is a well-established geriatric syndrome, the remaining LUTS share many risk factors and downstream consequences. In this symposium, each session will review the pre-clinical, epidemiologic, and clinical evidence supporting the entire umbrella of male and female LUTS to be considered a geriatric syndrome. Dr. Parker-Autry will describe the rationale and proposed approach for a multi-modal clinical care pathway to treat female geriatric incontinence syndrome that she has begun to implement in her academic geriatric urogynecology clinic. Dr. Al-Naggar then will review how biological hallmarks of aging contribute to LUTS and key considerations for pilot studies of gerotherapeutic interventions targeting urologic conditions based on a randomized, placebo-controlled clinical trial testing MitoQ for overactive bladder in older women with metabolic syndrome. Lastly, Dr. Bauer will review the evidence of male LUTS as a geriatric syndrome and will describe how this novel framework led to a R01-funded clinical trial comparing a remote exercise coaching intervention versus health education control for physically inactive older men with LUTS.

